# Experimental pilot study for augmented reality-enhanced elbow arthroscopy

**DOI:** 10.1038/s41598-021-84062-7

**Published:** 2021-02-25

**Authors:** Michiro Yamamoto, Shintaro Oyama, Syuto Otsuka, Yukimi Murakami, Hideo Yokota, Hitoshi Hirata

**Affiliations:** 1 Department of Hand Surgery, Nagoya University Graduate School of Medicine, 65 Tsurumai-cho, Showa-ku, Nagoya, 466-8550 Japan; 2 Department of Mechanical Engineering, Tokyo University of Science, Noda, Japan; 3 Image Processing Research Team, RIKEN Center for Advanced Photonics, Wako, Japan

**Keywords:** Medical research, Engineering

## Abstract

The purpose of this study was to develop and evaluate a novel elbow arthroscopy system with superimposed bone and nerve visualization using preoperative computed tomography (CT) and magnetic resonance imaging (MRI) data. We obtained bone and nerve segmentation data by CT and MRI, respectively, of the elbow of a healthy human volunteer and cadaveric Japanese monkey. A life size 3-dimensional (3D) model of human organs and frame was constructed using a stereo-lithographic 3D printer. Elbow arthroscopy was performed using the elbow of a cadaveric Japanese monkey. The augmented reality (AR) range of error during rotation of arthroscopy was examined at 20 mm scope–object distances. We successfully performed AR arthroscopy using the life-size 3D elbow model and the elbow of the cadaveric Japanese monkey by making anteromedial and posterior portals. The target registration error was 1.63 ± 0.49 mm (range 1–2.7 mm) with respect to the rotation angle of the lens cylinder from 40° to − 40°. We attained reasonable accuracy and demonstrated the operation of the designed system. Given the multiple applications of AR-enhanced arthroscopic visualization, it has the potential to be a next-generation technology for arthroscopy. This technique will contribute to the reduction of serious complications associated with elbow arthroscopy.

## Introduction

Available evidence supports the use of elbow arthroscopy to manage multiple conditions including rheumatoid arthritis, osteoarthritis, tennis elbow, and osteochondritis dissents. A major drawback of elbow arthroscopy is the risk of intraoperative complications, including serious neurovascular injuries^1^. The small working space and near adjacency of neurovascular and arthroscopic portals make elbow arthroscopy a technically demanding procedure. Successful elbow arthroscopy requires extensive knowledge of the spatial correlations among the neurovasculature, entry portals, and joint structures.

Recent advancements in sophisticated image processing technology have made precise preoperative simulations a possibility, and they are becoming increasingly common in clinical practice^2^. However, this valuable set of information is ineffectively utilized in elbow arthroscopy at arguably the most decisive point: during the procedure^3^. The ability to access such data that is optimized for use and seamlessly integrated into the surgical navigation system has remained elusive. We propose that the safety of standard elbow arthroscopy can be improved by incorporating augmented reality (AR). AR can allow the delivery of selective complex and highly useful information through computer graphics (CG) superimposed onto real-time video.

AR-assisted surgery is expanding rapidly in the field of sports, trauma, oncology, spine, and arthroplasty^4,5^. AR is an important technology because it enables the demonstration of the positional relationship between implants and targeted tissues to help complex interventions. However, AR-assisted arthroscopy has only few reports including knee and wrist arthroscopy^6,7^. Neurovascular injury is less significant with knee and wrist arthroscopy, because important neurovascular structures do not run near the portal or in the surgical field. Therefore, previous AR arthroscopy reports of the knee and wrist provided only superimposed bone and joint 3D data. On the other hand, all nerves around the elbow are at risk of injury during arthroscopy. Development of a novel technology using AR for elbow arthroscopy is needed with superimposed bone and nerve visualizations based on computed tomography (CT) and magnetic resonance imaging (MRI) data.

The purpose of this study was to develop and evaluate a novel elbow arthroscopy system that uses AR technology to superimpose nerve data on an arthroscopy monitor. We hypothesize that the accuracy of the resulting AR enhancement to standard arthroscopy would be acceptable.

## Methods

### Experiment 1

#### Data collection, processing and 3D modeling of body organs

Skin, bone, and nerve segmentation data of the elbow of a healthy human volunteer was obtained by CT and MRI. Inter-modal voxel registration was performed using ANT software with a SyN non-linear registration algorithm and affin registration^8^. Segmentation and refinement were performed using VoTracer software (Riken, Wako, Japan, http://www.riken.jp/brict/Ijiri/VoTracer/)^9^. All segmented lesion data were exported as standard triangulated language (STL) data.

**Figure 1:**
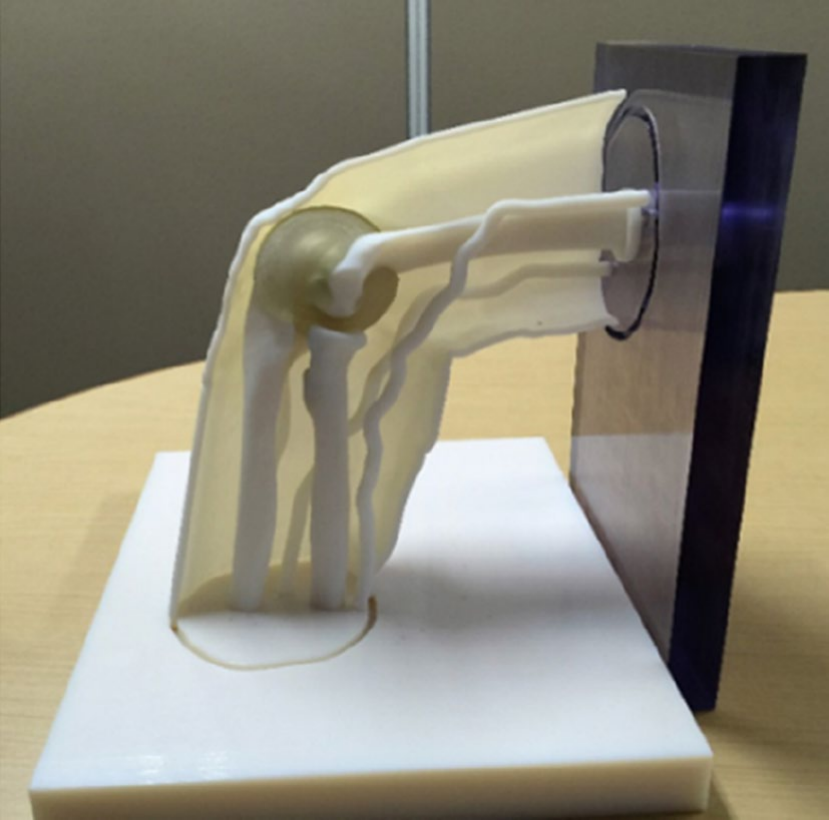
A real size 3-dimensional (3D) model of organs and frame. The model was constructed using a standard triangulated language (STL) 3D printer (Object500 Connex, Stratasys Ltd, US.).

We added support frame STL data to correctly coordinate bones and nerves upon 3D printing and printed a life-size 3D model of organs and frame using a stereo-lithographic 3D printer (Object500 Connex, Stratasys Ltd, US.) (Fig. 1).

#### Setup of elbow arthroscopy and device tracking system

We used a tracking system (MicronTracker3 H3-60 model; ClaroNav, Toronto, Canada) for surgical device tracking. MicronTracker3 is an optical pose tracking system with a unique ability to track unlimited number of tools simultaneously.

Each tracking marker used in the system was composed of black and white regions, and the system computed target locations at the intersection of four high-contrast regions. Each of the four black and white boundary lines independently served to pinpoint the location of targets called ‘Xpoints’.

Unlike bright spot markers, Xpoints have information on location and orientation. This additional discriminating characteristic reduces erroneous mismatches between targets on the left and right images. It also reduces marker misidentification, as matching the characteristics of the observed targets against templates leads to identification. As misleading bright reflection spots are more common in an operating environment compared with Xpoints, the use of Xpoints reduces the risk of misidentification.

**Figure 2:**
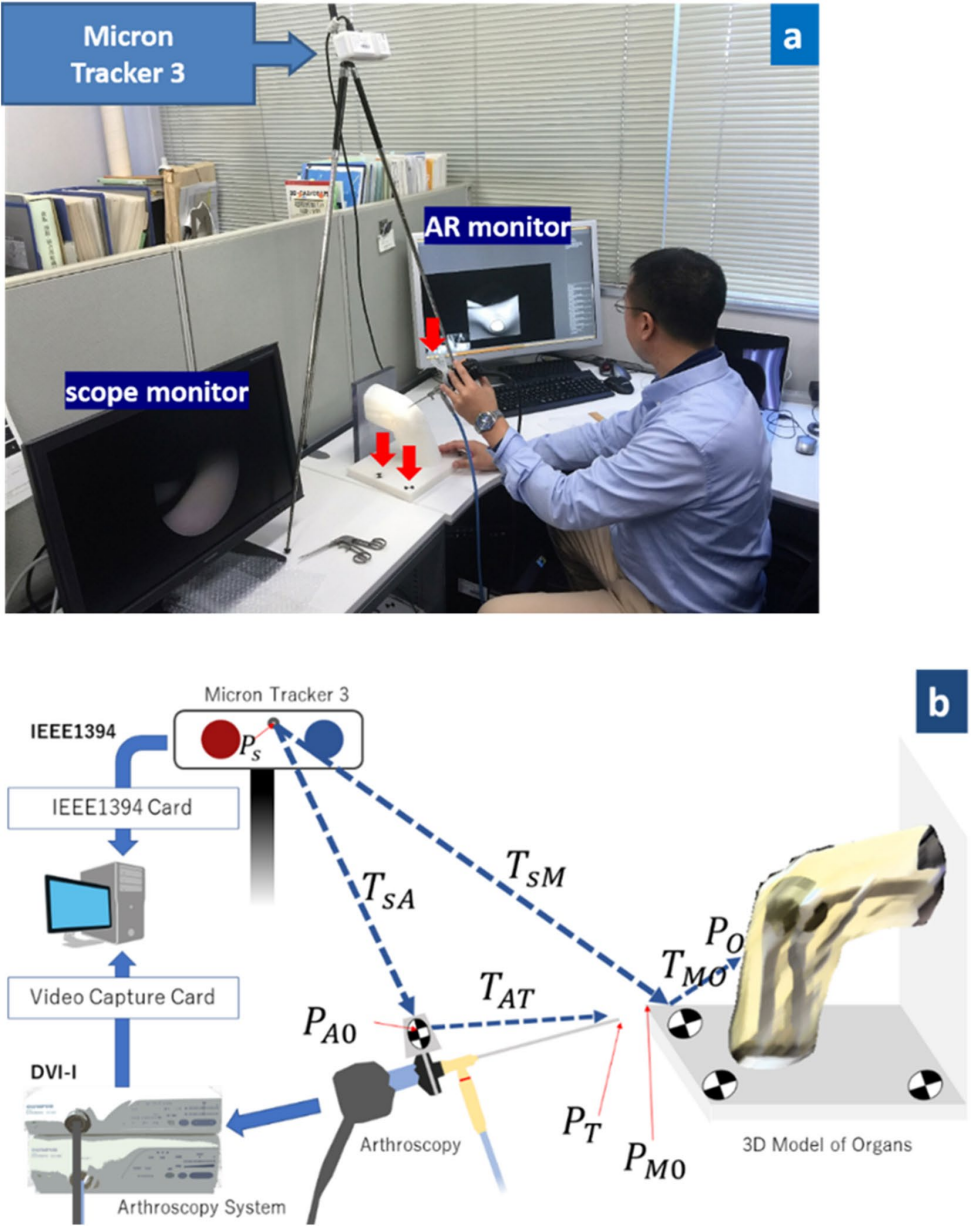
Elbow arthroscopy and tracking device system. Red arrows indicate Xpoints (**a**) The Schema of augmented reality (AR) arthroscopy system. This figure was designed by Dr. Shintaro Oyama using Adobe Illustrator CS5 software (https://www.adobe.com/jp) (**b**).

The markers were identified with reference to a marker template database and allowed to distinguish between multiple different instruments. Furthermore, the database can be updated during run-time, allowing new marker templates to be added by presenting them to the camera and assigning names to them. We placed different markers onto each 3D model baseplate and used the arthroscopy camera for tracking. To stabilize markers on the arthroscopy camera, we made custom stainless-steel guides that could attach markers on the arthroscope with a 30° angled lens (Fig. 2a).

#### Augmented reality image processing during training surgery

While performing elbow arthroscopy on the generated 3D model, an AR-calculated CG image was superimposed onto the arthroscopic video view by our AR system.

The system summary is as follows (Fig. 2b).

1. Arthroscopy image data was captured on the computer through a digital video capture card connected to the arthroscopy camera system.
2. The data of the 3D model base plate and the arthroscopy camera body loci were provided by MicronTracker3, which was able to trace target information using a customized software developed using the MicronTracker software developers’ kit. All the following transformation matrices T_ij_ from ${P}_{iO}({x}_{i},{y}_{i},{z}_{i})$ to ${P}_{jO}({x}_{j},{y}_{j},{z}_{j})$ are a 3 × 3 vector matrix in 3D real vector space.

The coordination system of the 3D model of organs and the arthroscopy camera were defined as Ʃ_M_ and Ʃ_A_. The transformation matrix from the MicronTracker3 sensor (${P}_{s}$) to the marker reference point (fiducial point) of Ʃ_M:__(P_M0_ = Ʃ_M (x, y, z = 0, 0, 0)_) and Ʃ_A:_ (P_A0_ = Ʃ_A (x, y, z = 0, 0, 0)_) was determined by stereo-triangulating the optical marker ${T}_{sM}$ and ${T}_{sA}$. The transformation matrix from ${P}_{M0}$ to each organ STL model reference point ${P}_{O}$ was pre-defined as ${T}_{MO}$ (although organ models include skin, radius, ulna, humerus, radial nerve, ulnar nerve, median nerve, musculocutaneous nerve, and they were handled separately in calculation, 3D relationships between these models were static; therefore, the reference points of these models were expressed as single points in this expression (${P}_{O}$), and ${P}_{A0}$ to the tip of arthroscopy light rod ${P}_{T}$ was pre-defined as ${T}_{AT}$ before examination.

3. Our custom-made software installed on the computer calculated each 3D organ model and arthroscopy light fiber rod position and direction. The position of the virtual camera was placed on the ${P}_{T}$ and rotated according to the lens-offset angle (in this experiment, it was 30°); therefore, the coordination system of camera sight Ʃ_c_ must consider this angle.

Calculation to transform Ʃ_M_ to Ʃ_c_ is as follows:

$$\sum_{c} = \, T_{AT} \sum_{A} = \, T_{sA} T_{sM}^{ - 1} \sum_{M}$$

Each 3D organ model data was rendered according to this transformation. A homogenous transformation can be constructed to register the virtual arthroscopy view to the real arthroscopy view. This calculation was performed with the assistance of OpenCV software (Intel, US).

4. The rendered image 3 was superimposed on the image 1 and displayed on the monitor.

#### Correction of barrel distortion in fisheye lens

**Figure 3:**
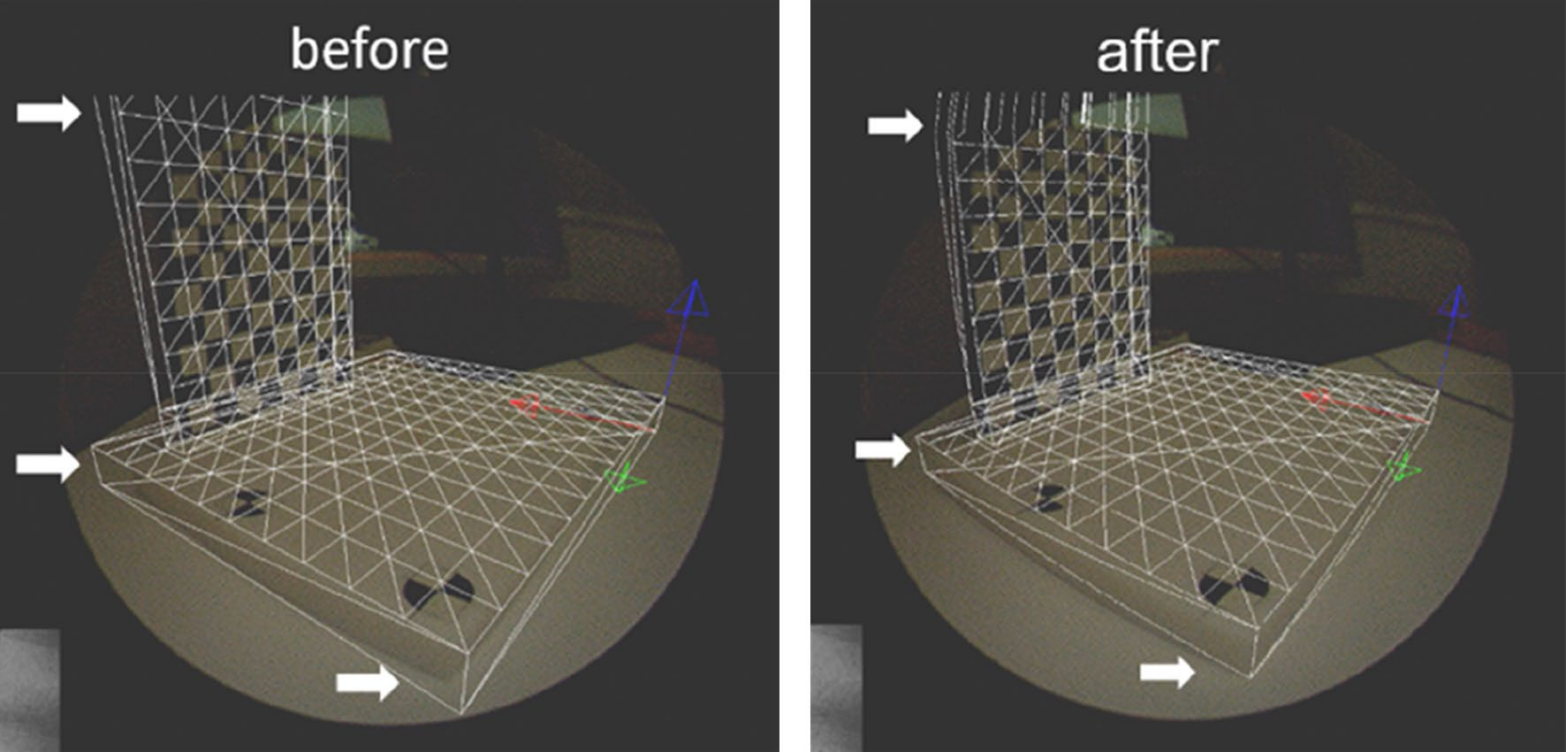
Reverse distortion correction using lens distortion matrix. White arrows show differences before and after correction. Appropriate distortion of the shape on the monitor was corrected.

The CG position and shape were initially different due to lens distortion. However, the CG position and shape were corrected to match the arthroscopic view using lens distortion parameters, which were estimated from the calibration pattern^10^. Reverse distortion correlation was performed using a lens distortion matrix. The matrix was pre-calculated using the calibration pattern of the arthroscopy camera (Fig. 3).

### Experiment 2

#### Data collection, processing and 3D modeling of organs

**Figure 4:**
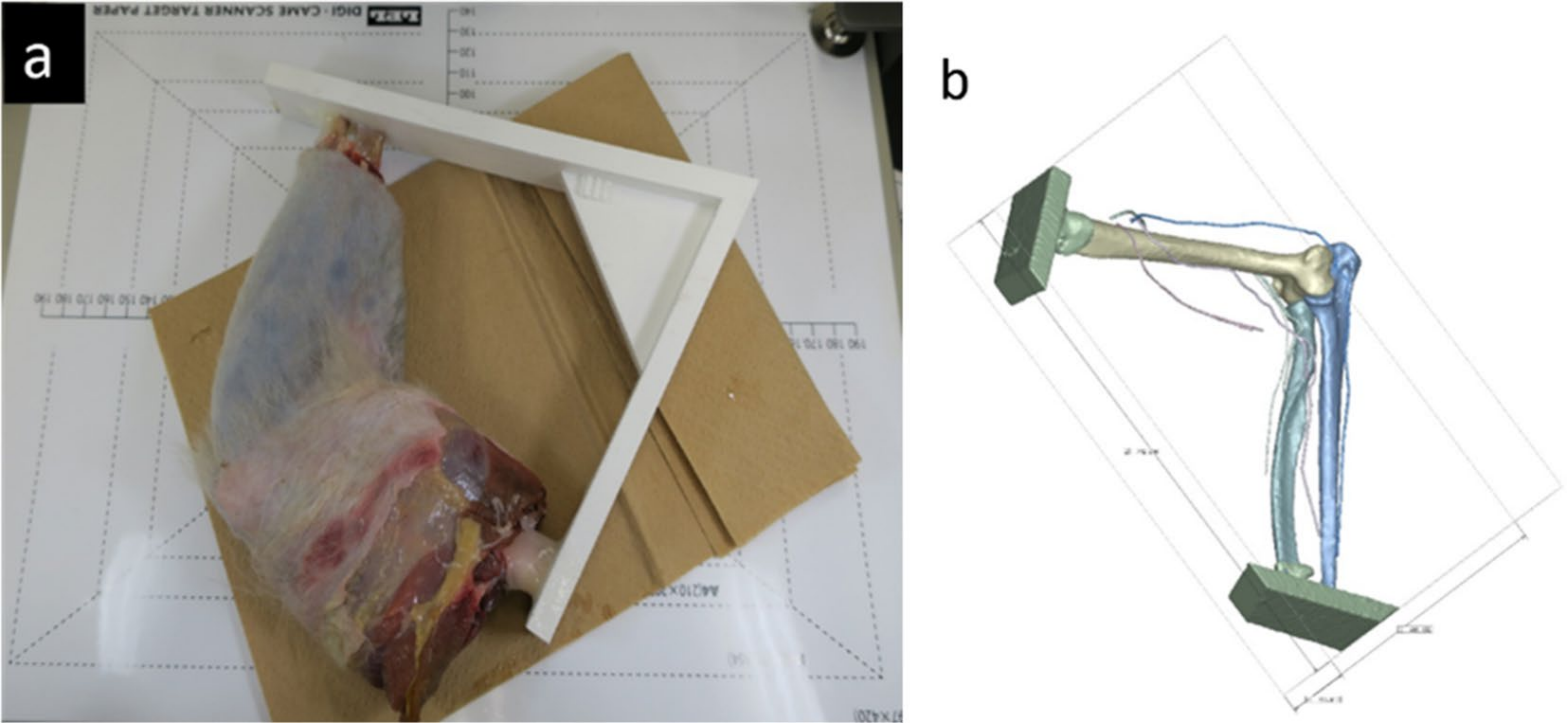
Elbow of a Japanese monkey cadaver and Standard Triangulated Language (STL) data. We used the elbow (1/2 of upper arm ~ 1/2 of forearm) of a Japanese monkey cadaver. From X-ray computed tomography (CT) data of the cadaveric elbow, we modeled frame data that can be precisely attached to humerus and ulna on the posture of 90° elbow flexion, 0° forearm pronation (**a**). We obtained bone segmentation from CT data and nerves from magnetic resonance imaging (MRI) data of a cadaveric Japanese monkey elbow. Segmentation and refinement were performed using VoTracer software (Riken, Wako, Japan) (http://www2.riken.jp/brict/Ijiri/VoTracer). This figure was generated by Dr. Shintaro Oyama using L-Phinus V5 Ver. 5.01 software (https://l-phinus.jp/software.html) (**b**).

Elbow (1/2 of upper arm ~ 1/2 of forearm) of a Japanese monkey cadaver was used for this experiment. The X-ray CT data of the cadaveric elbow were used with modeled frame data that could be precisely attached to the humerus and ulna on the posture at 90° elbow flexion and 90° forearm pronation. The frame was printed on a 3D printer (Davinci 1.0A / XYZ Printing, Inc. US.) using acrylonitrile butadiene styrene plastic. The frame was then fixed to the cadaver elbow with epoxy resin to ensure that it could not be easily moved (Fig. 4a).

X-ray CT and MRI of the elbow and frame were performed, and these datasets were used to obtain the bone and nerve data using methods similar to those used in experiment 1. We obtained bone segmentation from the CT data and nerve segmentation from the MRI data of a cadaveric Japanese monkey elbow. Segmentation and refinement were performed using VoTracer software (Riken, Wako, Japan). All segmented lesion data were exported as STL data (Fig. 4b).

#### Setup of elbow arthroscope and device tracking system

The tracking system setup was similar to experiment 1 except that we added an anti-pollution barrier to the system. A washable stainless-steel base plate was constructed to stabilize the elbow frame and placed at different markers on the baseplate and the arthroscopy camera head. The relative position between the baseplate and elbow frame was static. The 3D model base plate and arthroscopy camera body loci data were provided by MicronTracker3. The rendered images were superimposed on the real-time view and displayed on the AR monitor.

#### Augmented reality image processing during training surgery

Elbow arthroscopy was performed on the monkey elbow through anteromedial and posterior portals. While operating on the cadaver elbow, the AR calculated C image was superimposed onto the arthroscopic video by the same method as described in experiment 1. Registration of the CG data positioning was adjusted manually using the shapes of the capitellum and radial head obtained from an arthroscopic view.

#### Accuracy evaluation of AR position during rotation of arthroscopy

The AR range of error was examined to evaluate the accuracy of the system. The surgeon usually uses an arthroscope with a 30° angled lens for elbow arthroscopy and obtains the field of view by rotating the lens. We investigated the extent of target registration errors during the rotation of the lens. By installing a marker on the lens cylinder in addition to the marker installed on the camera head of the elbow arthroscope, the coordinate transformation matrix from the marker installed on the camera head to the marker on the lens cylinder of the arthroscope estimates the rotation angle around the axis. By estimating the direction of the elbow arthroscope from the estimated rotation angle, a 3D model that follows the rotation of the lens cylinder is superimposed.

**Figure 5:**
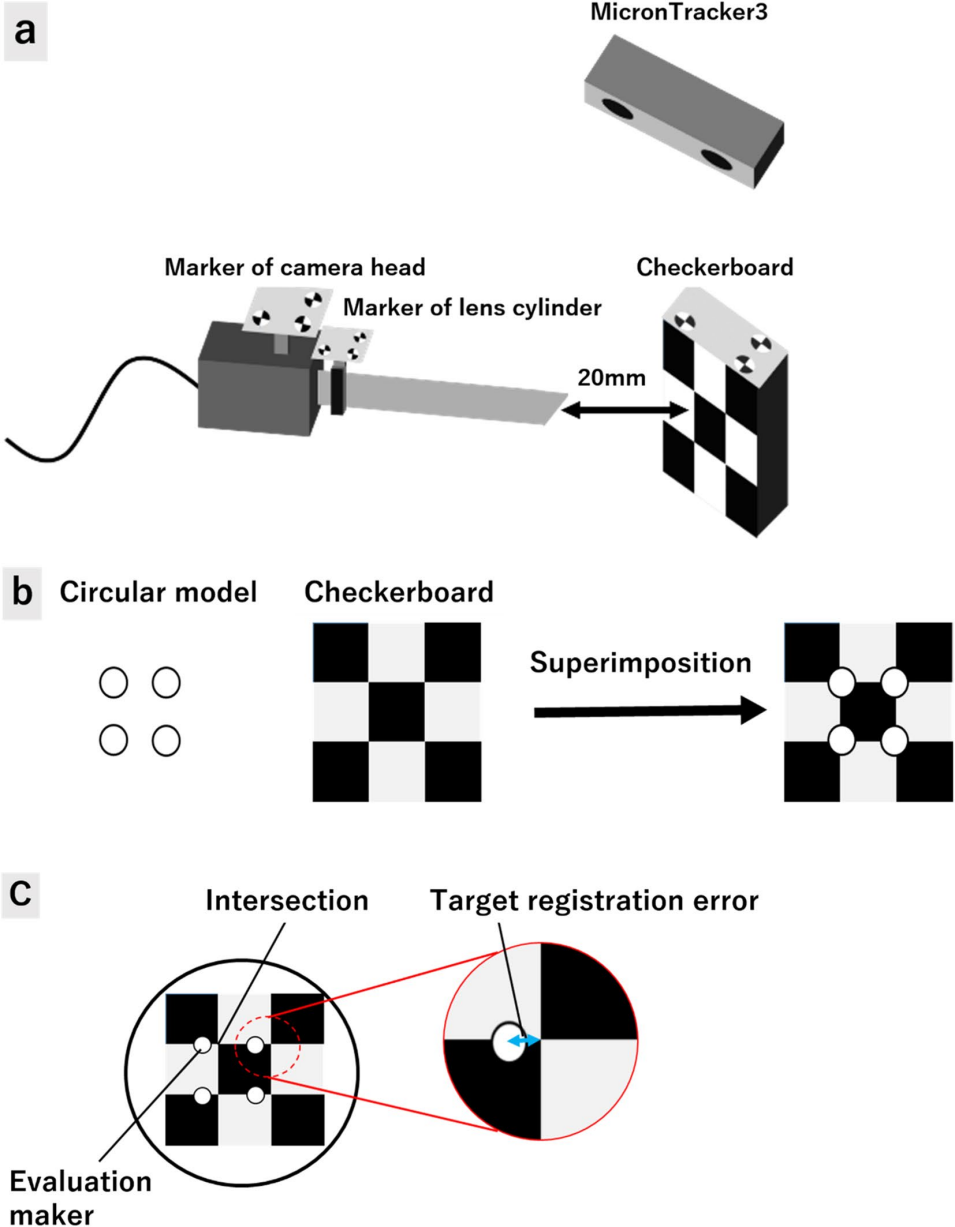
Augmented reality (AR) system error calculation during arthroscopy rotation. Markers were installed on the camera head and lens cylinder. The working distance was set to 20 mm (Fig. 5a). A circular model with a diameter of 2 mm was used as the 3D model, and a checkerboard was used as the object (Fig. 5b). The target registration error was obtained from the superimposed display to evaluate the position accuracy. The angle of the lens cylinder was changed by 10° within the measurable range (-40° < θ < 40°) of MicronTracker 3 (Fig. 5c). Figure 5a–c were designed by Syuto Otsuka using PowerPoint for Microsoft 365.

Markers were installed on the camera head and lens cylinder. The working distance was set to 20 mm to superimpose the 3D model using an elbow arthroscope whose various parameters were estimated by camera calibration (Fig. 5a).

A circular model with a diameter of 2 mm was used as the 3D model, and a checkerboard was used as the object. It was designed such that the center of the circle of the circular model and the intersection of the checkerboard overlap if there is no error in the superimposed position (Fig. 5b).

The target registration error was obtained from the superimposed display to evaluate the position accuracy. The angle of the lens cylinder was changed by 10° within the measurable range (− 40° < θ < 40°) of MicronTracker3 (Fig. 5c).

### Ethics approval and consent to participate

The study protocol was approved by the Ethics Committee of Nagoya University Hospital (2020-0013). Informed consent was obtained from all participants in this study. All methods in this study were performed in accordance with relevant guidelines and regulations.

## Results

### Experiment 1

We successfully performed AR arthroscopy for the full-size 3D elbow model. The CG data was superimposed onto the elbow arthroscopy video in real-time. We performed a registration to co-visualize the image of the patient’s elbow structures and the CG made by preoperative images. After manual modification of the position, scale, and orientation, the accuracy of the superimposed CG data was deemed acceptable on the AR monitor.

### Experiment 2

**Figure 6:**
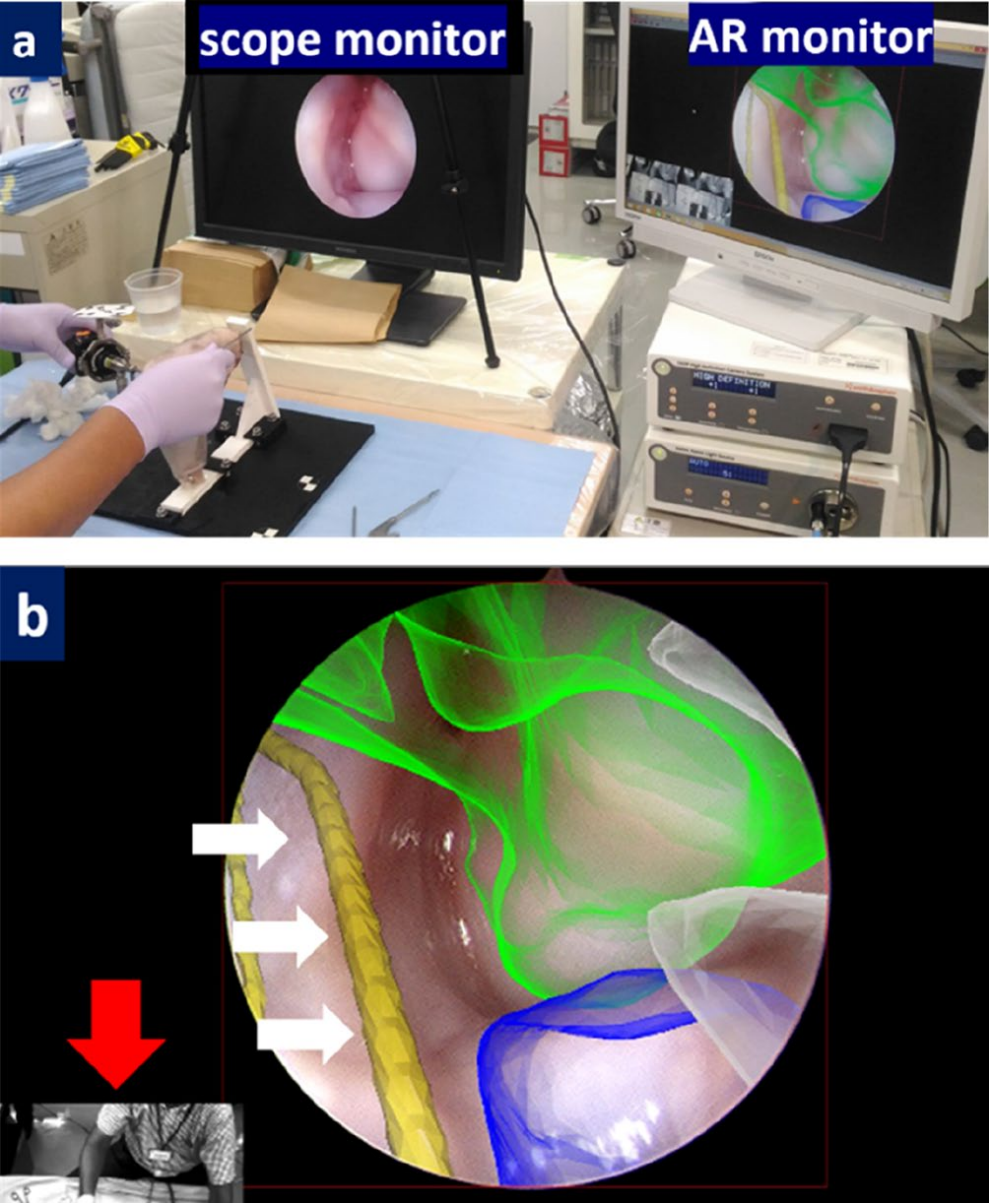
Augmented reality (AR) arthroscopy on cadaveric Japanese monkey elbow. Capitellum and radial head were visualized through the anteromedial portal and visualized on the scope monitor (**a**). The humeroradial joint and radial nerve (white arrows) were superimposed on the real view (**b**). The red arrow indicates a third person view using the stereo camera on the optical tracking device.

AR arthroscopy of the cadaveric Japanese monkey elbow was performed (Fig. 6a). The humeroradial joint and radial nerve were superimposed on the real-time view and displayed on the AR monitor. Although the radial nerve was not seen on the scope monitor as it was located behind the joint capsule, the position of the radial nerve was clearly observed. This was helpful to the surgeon in creating a lateral portal, thereby avoiding radial nerve injury (Fig. 6b).

### Accuracy evaluation of AR position during rotation of arthroscopy

**Figure 7:**
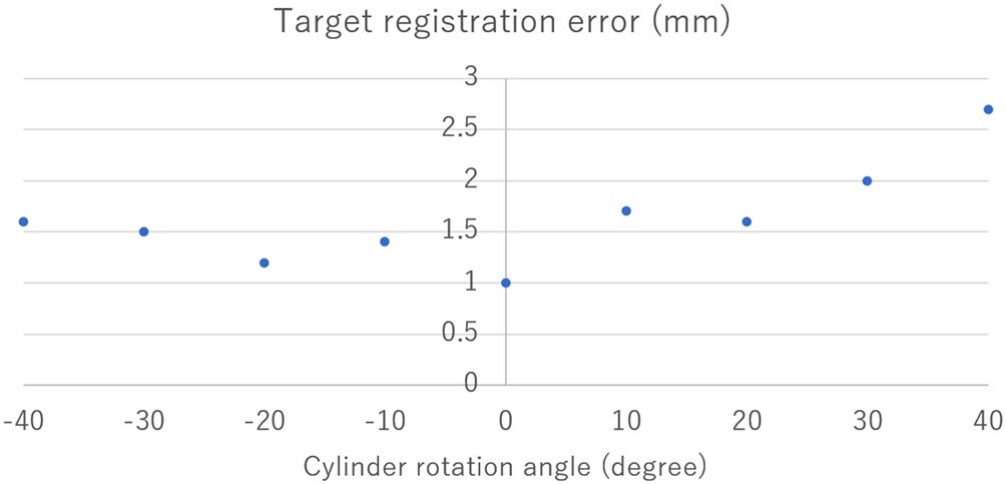
The target registration error. The target registration error was 1.63 ± 0.49 mm (range, 1–2.7 mm) with respect to the rotation angle of the lens cylinder from 40° to − 40°.

The target registration error was 1.63 ± 0.49 mm (range 1–2.7 mm) at a 20 mm scope-object distance, with respect to the rotation angle of the lens cylinder from 40° to − 40° (Fig. 7).

## Discussion

We integrated AR technology into elbow arthroscopy. We have demonstrated that the workings of the system and the accuracy of this AR system were deemed satisfactory. Through further iterations and refinements, AR-enhanced arthroscopic visualization has the potential to be a transformative technology. This technique will contribute to reducing the risk of serious complications associated with elbow arthroscopy.

The rapid development of endoscopy has enabled minimally invasive surgeries. However, this technique has a spatial perception disadvantage. The surgeon needs to alternate between the macroscopic view of the surgical field and the endoscopic view. AR navigation has recently been employed during brain, spinal, plastic, maxillofacial, and several other highly technically demanding surgeries^11–13^. However, few studies have focused on its use in upper limb arthroscopy^7^. There is an unmet need for the next-generation arthroscopy system especially designed for the elbow because of the high incidence of associated intraoperative complications.

Creating AR-enhanced navigation requires 3D preoperative imaging of the target tissue, AR display, tracking system, and a software to calculate the arthroscopy position and direction for each 3D organ.

VoTracer is a software employed for volume computer-aided design (VCAD) of pre-operative CT and MRI data. Segmentation and refinement of bone and nerve data can be performed using this software. A limitation associated with all VCAD software programs is the need for manual work in creating CG of the target tissue. Fine anatomical knowledge of the elbow, especially of the nerve route, is required to complete segmentation and refinement of the tissues. The technology for extracting and superimposing information on large skeletons in the hip and shoulder joints has already been established, and clinical application has advanced^14,15^. However, no attempt has been made to create a 3D CG that includes information on nerves with a diameter of approximately 10 mm around the elbow joint and display it on an arthroscopy monitor. The highlight of the current study is that elbow arthroscopic surgery, which causes many complications of nerve damage, will be transformed into safe surgery by this technology.

There are several methods of display for AR. See-through glasses and 3D projection mapping are possible AR displays. See-through glasses have a drawback in that it is difficult to obtain an accurate AR view superimposed on the real view. See-through glasses need to track the pupil positions in real-time for registration. 3D projection mapping is another way to display the AR view. In order to obtain an AR view on the patient’s skin, the video projector has to be set over the patient in the operating room. As both deep and superficial structures are displayed on the skin surface, a significant error of perception is noted when more than two surgeons see the AR display^11^. We employed a video-based display with two monitors for real and AR-enhanced views. This system was a natural fit for arthroscopy, as the surgeon could simultaneously confirm the real and AR-enhanced view. In this system, the surgeon can select to watch the real view or AR-enhanced view depending on the situation. However, in another AR study, fewer gaze shifts reduced task completion time and errors^16^. In the near future, it is necessary to develop a system that suppresses the gaze shifts of surgeons using fewer monitors with higher precision AR using real-time data.

A variety of tracking systems are available for clinical settings, such as infrared camera-based tracking, the tag video tracking, and electromagnetic tracking^12,17,18^. The accuracy of the tracking device is very important in the clinic as it is directly linked to safety. We used an optical tracking device, MicronTracker3. It was able to trace each target information in real-time using a customized software developers’ kit. The accuracy of this tracking system was deemed acceptable, and the position error was 1.63 ± 0.49 mm (range 1 to 2.7 mm) at a 20 mm scope-object distance, while rotation of the arthroscopy was 40° to − 40°. The error when the rotation angle of the lens cylinder is 0° represents the cumulative error other than the rotation angle estimation error, such as the parameter estimation that occurs during camera calibration and the estimation of positional orientation by MicronTracker3. Whether this accuracy is sufficient for clinical application needs to be confirmed in future clinical studies after further ensuring safety.

Arthroscopy simulator training improves the performance of students and residents during knee and shoulder surgery^19–23^. Recently, multiple types of virtual reality-based training simulators for arthroscopy have been reported^22^. Among these simulators, high-fidelity virtual reality simulation was reported to be superior to the low-fidelity model to acquire arthroscopic skills^23^. An AR-enhanced arthroscopic system with superimposed tasks can be a high-fidelity training tool for surgical education. This system can also provide a third-person view using the stereo camera on the optical tracking device, MicronTracker3. The third-person view and record of tracking makers provide a trainee feedback regarding the handling of scope and other instruments during surgery.

AR-enhanced navigation for arthroscopy may become the next generation arthroscopy system. However, this study has some limitations. First, we used preoperative imaging techniques, such as CT and MRI, but not real-time information of the target tissue. The size and location of the lesion at the time of surgery may differ from the preoperative data. Second, the elbow flexion angle was fixed in our experiments; however, surgeons in a clinical setting typically move the elbow during arthroscopy. Superimposed CG data, therefore, needs to change according to the elbow angle. AR with real-time data of the target tissue is required to solve these problems. Intraoperative CT, MRI, or ultrasonography may be employed to obtain intraoperative data of the target tissue. Actually, nerves around the elbow can be clearly visualized using ultrasonography^24,25^. In addition, an algorithm for intraoperative data is required.

## Conclusions

The technological integration of AR with arthroscopy was successful. We attained satisfactory accuracy and demonstrated the working of such a system. Upon resolution of some limitations, AR-enhanced arthroscopic visualization has the potential to become the next-generation arthroscopy. Elbow arthroscopy requires significant training for surgeons, and even skilled surgeons have reported complications during surgery. We believe that AR-enhanced arthroscopy will reduce the risk of serious complications associated with elbow arthroscopy.

## Data Availability

The datasets during the current study are available from the corresponding author on reasonable request.

## Abbreviations

AR: Augmented reality
CT: Computed tomography
MRI: Magnetic resonance imaging
3D: 3-Dimensional
CG: Computer graphics
STL: Standard triangulated language
VCAD: Volume computer aided design
