# Author Correction: Experimental pilot study for augmented reality-enhanced elbow arthroscopy

**DOI:** 10.1038/s41598-021-90073-1

**Published:** 2021-05-17

**Authors:** Michiro Yamamoto, Shintaro Oyama, Syuto Otsuka, Yukimi Murakami, Hideo Yokota, Hitoshi Hirata

**Affiliations:** 1 Department of Hand Surgery, Nagoya University Graduate School of Medicine, 65 Tsurumai-cho, Showa-ku, Nagoya, 466-8550 Japan; 2 Department of Mechanical Engineering, Tokyo University of Science, Noda, Japan; 3 Image Processing Research Team, RIKEN Center for Advanced Photonics, Wako, Japan

Correction to: *Scientific Reports* 10.1038/s41598-021-84062-7, published online 25 February 2021

This Article contains an error where the Acknowledgements section was omitted. The Acknowledgements section should read:

“We thank Takehiro Tawara PhD, who belonged to the Image Processing Research Team, RIKEN Center for Advanced Photonics at the time of this research, for his contribution.”

